# Global stability analysis of hepatitis B virus dynamics

**DOI:** 10.12688/f1000research.52785.1

**Published:** 2021-05-28

**Authors:** Olajumoke Oludoun, Olukayode Adebimpe, James Ndako, Oluwakemi E. Abiodun, Babatunde Gbadamosi, Benedicta B. Aladeitan

**Affiliations:** 1 Department of Physical Sciences, Landmark University, Omu-Aran, Kwara State, Nigeria; 2 Department of Microbiology, Landmark University, Omu-Aran, Kwara State, Nigeria; 3 Department of Computer Sciences, Landmark University, Omu-Aran, Kwara State, Nigeria

**Keywords:** Hepatitis B, mathematical model, positivity and existence, global stability, sensitivity, Lyapunov method, simulation

## Abstract

This paper considers the impact of an acute individual's spontaneous clearance, recovery of a chronic individual with full immunity, and risk factor reduction on a hepatitis B virus (HBV) model. The existence and the positivity solution of the model are established. The model threshold quantity is defined and sensitivity analysis is analyzed to demonstrate the effect of various parameters on the spread of the virus. The global stability analysis of the equilibrium is shown using Lyapunov and comparison theorem methods. Finally, computational simulation is presented to validate the analytical solution. The results show that treatment, spontaneous clearance and reduction of the risk factor are highly successful in transmitting and regulating HBV transmission. The effective measure of these parameters as substantiated by our simulations, providing an excellent control method of the transmissible infection of HBV.

## Introduction

Hepatitis B is a common liver infection caused by the potentially life-threatening hepatitis B virus (HBV). HBV can cause a serious infection, which places individuals at high risk of dying from fibrosis and cirrhosis of liver. It is a huge worldwide health issue. As reporting by the World Health Organization, around 360 million of the 2 billion people infected with the HBV are reported to have a lifelong chronic infection, and 887,000 of those individuals die from liver cirrhosis or primary hepatocellular carcinoma ( WHO, 2020). As of 2016, 27 million individuals (10.5%) of all people considered to be living with HBV were aware of their infection, while 4.5 million (16.7%) of those diagnosed were receiving treatment ( WHO, 2019). The Western Pacific region recorded the highest incidence rate of HBV at 6.2% of the adult population, while this was 6.1% in the African region, and 0.7% in the American region on ( WHO, 2019). Although HBV lives outside the body for about seven days, it is still very possible for it to cause an infection if it is injected into an unvaccinated individual. It takes about 75 days on the average for the HBV to incubate but this can vary between 30 and 180 days. Detection of the virus can be between 30 and 60 days of being infected or consequently mature into full-blown HBV ( CDC, 2019).

The HBV, a hepatotropic non-cytopathic virus, is responsible for the disease ( Ribiero, 2002). In highly endemic areas, perinatal transmission or horizontal transmission (exposure to infected blood) are the primary means of transmission ( Pan and Zhang, 2005). The most common method of transmission is from mother to child at birth, particularly from infected children to uninfected children within the first 5 years of life. Contaminated body fluids such as vaginal discharge, saliva, menstrual flow, and semen are other means of transmission ( Pan and Zhang, 2005). Rarer means of transmission include transpiration, breast milk, sweat, and urine by percutaneous or mucosal exposure of infected individuals ( Mpeshe and Nyerere, 2019). In particular, unvaccinated men who have sex with men and heterosexual people with several sexual partners or who have contact with sex workers may experience sexual transmission of HBV ( Khan et. al, 2019). In less than 5% of cases, infections lead to chronic hepatitis in adulthood. Virus transmission can also occur either in health care facilities or through the reuse of needles and syringes among individuals who inject drugs. Furthermore, infection can occur during medical, surgical and dental procedures, by tattooing, or by using razors and similar products contaminated with infected blood ( Mpeshe and Nyerere, 2019).

Typically, there is a 5-10% chance of recovery for adults that develop chronic infections ( Chenar et al., 2018). Host variables are believed to be responsible for determining whether the infection is cleared or becomes chronic, especially immune responses ( Ciupe et al., 2007). Different aspects of HBV dynamics and the immune response during infection have been investigated by several mathematical models ( Ribiero, 2002; Long et al., 2008; Lau et al., 2009; Wang et al., 2010; Qesmi et al., 2010; Pang et al., 2010).

As a result of the research mentioned above, we present an infectious disease model to better understand how testing and treatment affects HBV transmission dynamics and prevalence. The model formulation of HBV transmission dynamics, as well as the dynamical behavior of the model, including equilibria and stabilities is presented in this paper. The aim of this study is to contribute to the development of HBV control strategies and the establishment of intermediate objectives for intervention programs.

## Model formulation

It has been clinically shown that a proportion of HBV acutely infected individuals can spontaneously clear the virus ( Pan and Zhang, 2005). Also, infectious individuals under treatment can become prone to re-infection if they discontinue treatment, or consume alcohol or use of drugs, which can reduce the impact of the treatment. In view of this, the following model is developed where the population is divided into different states, namely: the susceptible, the acute, the chronic carriers, the treated and the recovered states.

At time t , denoted by $N\left(t\right),$ the total population is divided into the following five classes/subgroups ( Table 1) corresponding to different epidemiological status.

(1) $$N\left(t\right)=S\left(t\right)+A\left(t\right)+C\left(t\right)+T\left(t\right)+R\left(t\right)\;$$

where $S\left(t\right)$ are the susceptible populace, $A\left(t\right)$ is the populace that are acutely infected with HBV, $C\left(t\right)$ are the chronically/clinically infected individuals, while $T\left(t\right)$ are individuals under treatment and $R\left(t\right)$ are the recovered classes.

**Table 1.** Parameter descriptions.

Parameter	Description
ζ	birth rate
α	Proportion of population successfully immunized
γ	Probability that children born to carrier mothers will develop to chronic state
$\lambda_{s}$	Horizontal transmission coefficient
ξ	Reduced transmission coefficient
η	Spontaneous clearance proportion
1ω	Duration of acute phase
k	Rate at which treated individuals recover with full immunity
μ	Natural death rate
σ	Treatment rate for chronic individuals
ν	Proportion of population recovering
ρ	Duration of HBV treatment
ε	Rate at which recovered population fall out from risk reduction
S(t)	Susceptible Compartment
A(t)	Acute Compartment
C(t)	Chronic Compartment
T(t)	Treatment Compartment
R(t)	Recovered Compartment

**Figure 1.**
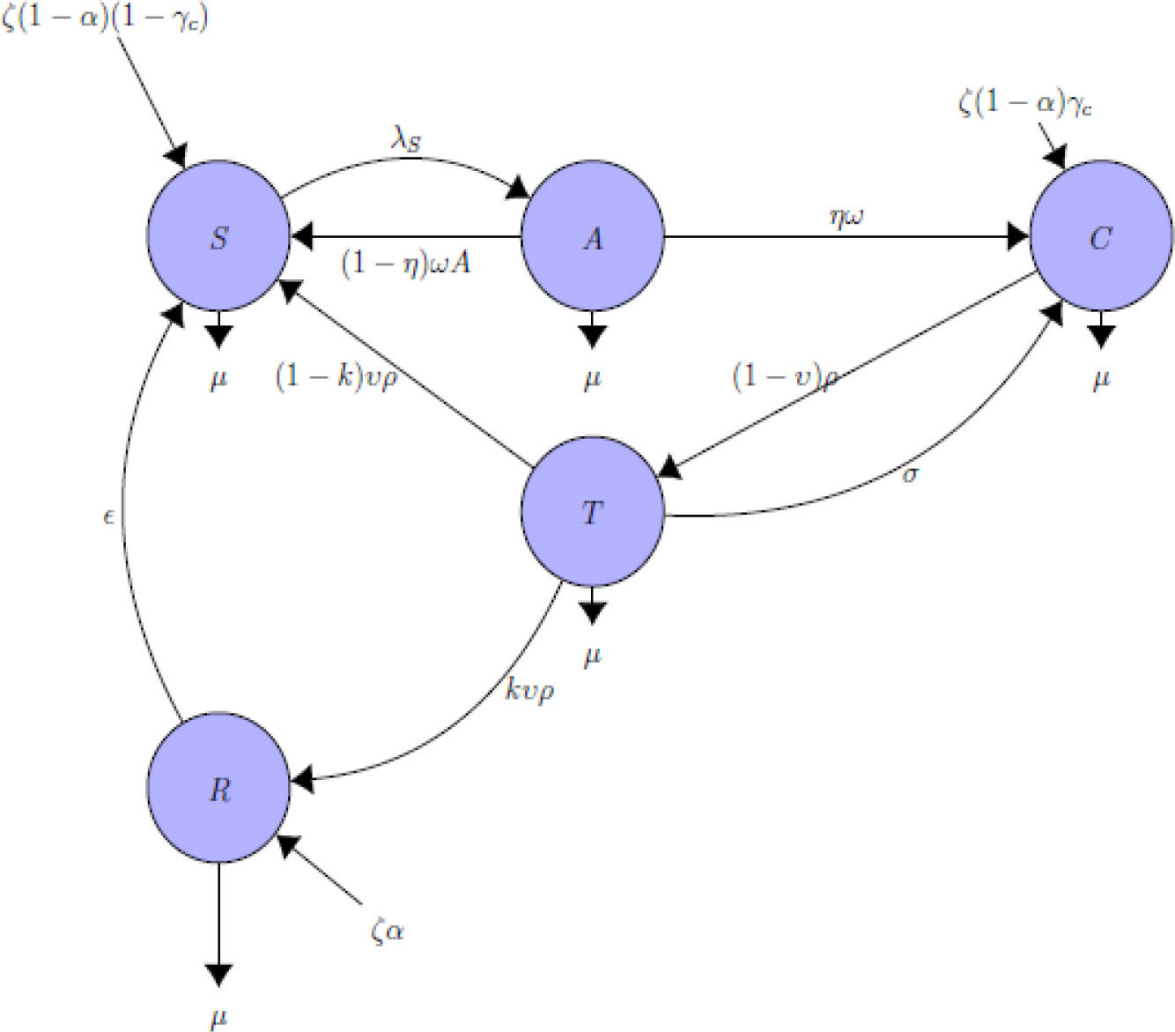
Compartmental flow diagram of hepatitis B virus.

Figure 1 schematically represents the epidemiology of HBV. The various disease stages are replicated by the various compartments (circle) and the arrows demonstrate the way an individual progress from one state to the other. It is assumed that at time t, susceptible individual S, enter the population at a constant rate $\;\zeta \left(1-\alpha \right)\left(1-\mathrm{\gamma C}\right)\;$ where ζ is the birth rate, α is the proportion of population successfully immunized, while γ is the probability that children born to carrier mothers will develop to chronic state. For all classes, individuals die at a constant natural mortality rate, μ. We assume that HBV infected individuals on treatment are infectious. Susceptible individual S may acquire HBV infection when in contact with individuals in A,C,andT populace at a rate $\lambda_{s}$ (force of infection associated with HBV), where

(2) $$\lambda_{s}=\mathrm{\beta A}+\xi_{1}\mathrm{\beta C}+\xi_{2}\mathrm{\beta T}$$

where βA and βC are the effective contact rate for HBV infection to occur/probability that a contact will result in an Acute and Chronic HBV compartment, respectively. Modification ξ>1 accounts for a higher risk of HBV acquisition for people living with Chronic HBV.

A proportion of the acute HBV infected individuals η, becomes chronic carriers and then get treated at σ, while the remaining proportion $\left(1-\eta \right)$ spontaneously clear the virus. $\frac{1}{\omega }$ is the duration of acute phase. A proportion of the treated HBV individuals, κ, recover with full immunity, some were in the process of recovering in the treated populace at a rate, υ and duration for the treatment is given as ρ while the remaining proportion $\left(1-\kappa \right)$ becomes susceptible. Those individuals in the process of recovering in the treated populace at a rate, υ if engaging/exposed to high-risk habit and those on treatment ρ can be re-infected at the rate νρ if they discontinue treatment at a rate of ε .

$$\frac{\mathrm{dS}}{\mathrm{dt}}=\zeta \left(1-\alpha \right)\left(1-\gamma C\right)-\lambda_{s}S+\left(1-\eta \right)\omega A-\mu S+\left(1-k\right)\nu \rho T+\varepsilon R$$

$$\frac{\mathrm{dA}}{\mathrm{dt}}=\lambda_{s}S-\omega A-\mu A$$

(3) $$\frac{\mathrm{dC}}{\mathrm{dt}}=\mathrm{\eta \omega A}+\zeta \left(1-\alpha \right)\mathrm{\gamma C}+\left(1-\upsilon \right)\mathrm{\rho T}-\mathrm{\sigma C}-\mathrm{\mu C}$$

$$\frac{\mathrm{dT}}{\mathrm{dt}}=\sigma C-\rho T-\mu T$$

$$\frac{\mathrm{dR}}{\mathrm{dt}}=\zeta \alpha +\mathrm{k\upsilon \rho T}-\varepsilon R-\mu R$$

where $\lambda_{s}=\mathrm{\beta A}+\xi_{1}\mathrm{\beta C}+\xi_{2}\mathrm{\beta T}$

## Model implementation

### Positivity and boundedness of solutions

From model (3), we observed that the variables are nonnegative and the solutions are non-negative for all time t≥0. The parameters used are assumed to be positive and show that the feasible solutions are bounded in the region.

*Lemma 1:* The initial values of the parameters are

$$\left\{S\left(0\right)\geq 0,A\left(0\right)\geq 0,C\left(0\right)\geq 0,T\left(0\right)\geq 0,R\left(0\right)\geq ,0\text{and}N\left(0\right)\geq 0\right\}\in \Phi$$

Then the solution of the model $\left\{S\left(t\right),A\left(t\right),C\left(t\right),T\left(t\right),R\left(t\right),N\left(t\right)\right\}$ is positive for all t≥0.

*Proof:* Considering the first equation in (3),

$$\frac{\mathrm{dS}}{\mathrm{dt}}=\zeta \left(1-\alpha \right)\left(1-\gamma C\right)-\lambda_{s}S+\left(1-\eta \right)\omega A-\mu S+\left(1-k\right)\nu \rho T+\varepsilon R$$

we have,

$$\frac{\mathrm{dS}}{\mathrm{dt}}\geq -\left(\lambda_{s}+\mu \right)S\int \frac{1}{S}\mathrm{dS}\geq \int -\left(\lambda_{s}+\mu \right)\mathrm{dt}$$

$$S\geq S_{0}e^{-\left(\lambda_{s}+\mu \right)}\geq 0$$

Hence, S≥0

With respect to the second equation in (3), we have

$$\frac{\mathrm{dA}}{\mathrm{dt}}=\lambda_{s}S-\omega A-\mu A$$

$$\frac{\mathrm{dA}}{\mathrm{dt}}\geq -\left(\omega +\mu \right)A$$

$$\int \frac{1}{A}\mathrm{dA}\geq \int -\left(\omega +\mu \right)\mathrm{dt}$$

$$A\geq A_{0}e^{-\left(\omega +\mu \right)}\geq 0$$

Hence, A≥0

The same approach applies to the proof of the positivity of C, T and R.

### Equilibrium points and reproduction number

The disease-free equilibrium of the model (3) exists and is given by:

(4) $$\left(E_{o}\right)=\left[\frac{\zeta \left(1-\alpha \right)}{\mu },0,0,0,0\right]\;$$

The endemic steady state of the model (3) exists and is presented as follows:

(5) $$S^{*}=-\left(\frac{\left(\mu +\omega \right)\left(\zeta \gamma \left(\mu +\rho \right)\left(\alpha -1\right)\rho \sigma \nu +\mu^{2}+\mu \rho +\mu \sigma \right)\varepsilon \left(\alpha \mu -\varepsilon -\mu \right)}{L}\right)$$

(6) $$A^{*}=\left(\frac{S^{*}}{\Lambda \left(\mu +\omega \right)}\right)$$

(7) $$C^{*}=-\left(\frac{\left(\mu +\rho \right)\text{ηωΛξ}\left(\alpha \mu -\varepsilon -\mu \right)}{L}\right)$$

(8) $$T^{*}=\left(\frac{C^{*}}{\sigma \left(\mu +\rho \right)}\right)$$

(9) $$R^{*}=\left(\frac{H}{L}\right)$$

where

**Figure:**
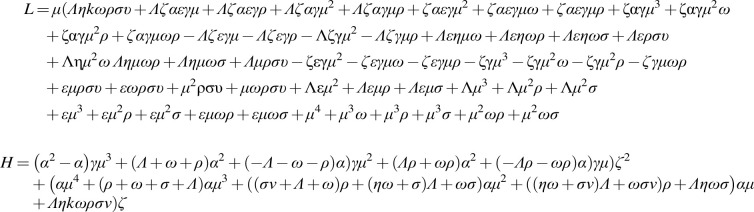


By using the next generation matrix, the basic reproduction number is determined and given by:

$$F=\left[\begin{array}{ccc} \frac{\beta \zeta \left(1-\alpha \right)}{\mu } & \frac{\xi_{1}\beta \zeta \left(1-\alpha \right)}{\mu } & \frac{\xi_{2}\beta \zeta \left(1-\alpha \right)}{\mu }\\ 0 & 0 & 0\\ 0 & 0 & 0 \end{array}\right]$$

$$V=\left[\begin{array}{ccc} \omega +\mu & 0 & 0\\ -\eta \omega & -\zeta \left(1-\alpha \right)\gamma +\sigma +\mu & 0\\ 0 & -\sigma & \rho +\mu \end{array}\right]$$

**Figure:**
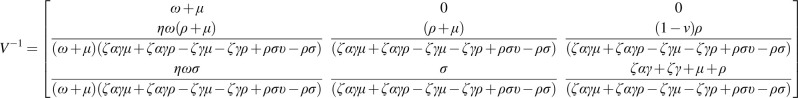


The reproduction number is given by *ρ*( *FV*−1), and

(10) $$R0=\frac{\beta \zeta \left(1-\alpha \right)}{\mu \left(\omega +\mu \right)}+\frac{\beta \zeta \left(1-\alpha \right)\eta \omega \left(\mu \xi_{1}+\rho \xi_{1}+\sigma \xi_{2}\right)}{\mu \left(\omega +\mu \right)\left(\text{ζαγμ}+\text{ζαγρ}-\zeta \gamma \mu -\zeta \gamma \rho +\rho \sigma \upsilon -\rho \sigma \right)}$$

### Global stability of the equilibria

The global stability of the disease-free equilibrium was investigated using the Comparison method at the disease - free equilibrium $E_{o}$ _._ Theorem 1 proves the global stability of disease -free equilibrium $E_{o}$

*Theorem 1:* The disease - free equilibrium $E_{o}$ of system (3) is globally asymptotically stable if $R_{o}<1$ and unstable if $R_{o}>1$ .

*Proof:* The Comparison method as implemented in Lashmkantham, *et al* (1989) and Mushayabasa *et al* (2011) is used here. The rate of change of the acute and chronic components of system (3) can be written as

$$\left(\begin{array}{c} \frac{\mathrm{dA}}{\mathrm{dt}}\\ \frac{\mathrm{dC}}{\mathrm{dt}}\\ \frac{\mathrm{dT}}{\mathrm{dt}} \end{array}\right)=\left(F-V\right)\left(\begin{array}{c} A\\ C\\ T \end{array}\right)-\left(1-\frac{S}{N}\right)$$

where,

$$F=\left[\begin{array}{ccc} \frac{\beta \zeta \left(1-\alpha \right)}{\mu } & \frac{\xi_{1}\beta \zeta \left(1-\alpha \right)}{\mu } & \frac{\xi_{2}\beta \zeta \left(1-\alpha \right)}{\mu }\\ 0 & 0 & 0\\ 0 & 0 & 0 \end{array}\right]$$

$$V=\left[\begin{array}{ccc} \omega +\mu & 0 & 0\\ -\eta \omega & -\zeta \left(1-\alpha \right)\gamma +\sigma +\mu & 0\\ 0 & -\sigma & \rho +\mu \end{array}\right]$$

Since at the disease free $A=C=T=R=0\to \left(0,0,0,0\right)$ and S≤N as t→∞ .

Thus,

$$\left(\begin{array}{c} \frac{\mathrm{dA}}{\mathrm{dt}}\\ \frac{\mathrm{dC}}{\mathrm{dt}}\\ \frac{\mathrm{dT}}{\mathrm{dt}} \end{array}\right)\leq \left(F-V\right)\left(\begin{array}{c} A\\ C\\ T \end{array}\right)$$

Then all eigenvalues of the matrix $\left(F-V\right)$ have negative real parts, i.e

$$\left[\begin{array}{ccc} \frac{\beta \zeta \left(1-\alpha \right)}{\mu }-\omega -\mu -\lambda & \frac{\xi_{1}\beta \zeta \left(1-\alpha \right)}{\mu } & \frac{\xi_{2}\beta \zeta \left(1-\alpha \right)}{\mu }\\ \eta \omega & \zeta \left(1-\alpha \right)\gamma +\sigma +\mu -\lambda & 0\\ 0 & \sigma & -\left(\rho +\mu \right)-\lambda \end{array}\right]=0$$

**(11):**
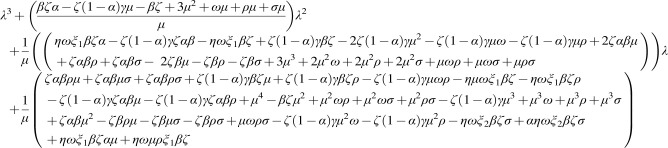


Equation (11) has three negative roots by Descartes rule of signs if

**Figure:**
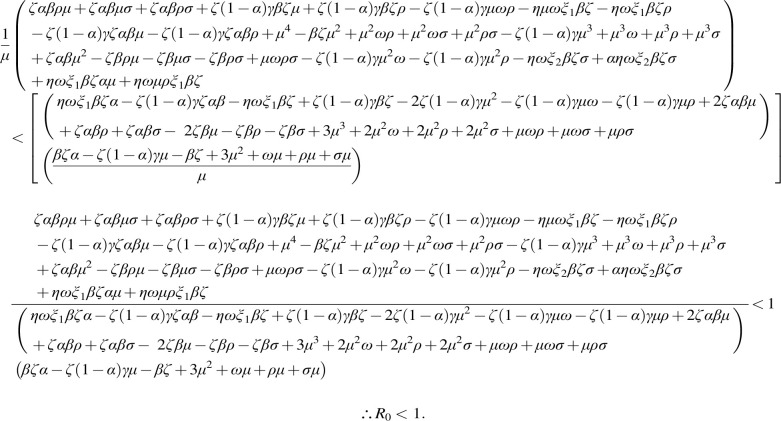


It follows that the linearized differential inequality is stable whenever $R_{0}<1$ . Consequently, $\left(A,C,T\right)\to \left(0,0,0\right)$ as t→∞ . Evaluating system (3) at A=C=T=0 gives S→1 for $R_{0}<1$ . Hence, the disease-free equilibrium $E_{0}$ of system (3) is globally asymptotically stable if $R_{0}<1$ . The result also follows immediately that the disease-free equilibrium $E_{0}$ of system (3) is unstable if $R_{0}>1$ .

*Theorem 2:* The equations of the model has a positive distinctive endemic equilibrium whenever R0 > 1, which is said to be globally asymptotically stable.

*Proof:* Considering the Lyapunov function defined as:

**(12):**
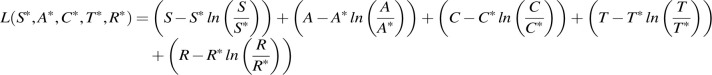


where L takes it derivative along the system directly as:

**(13):**
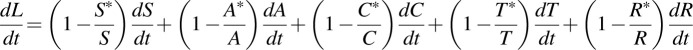


**(14):**
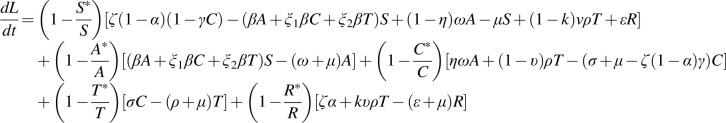


At equilibrium,

**(15):**
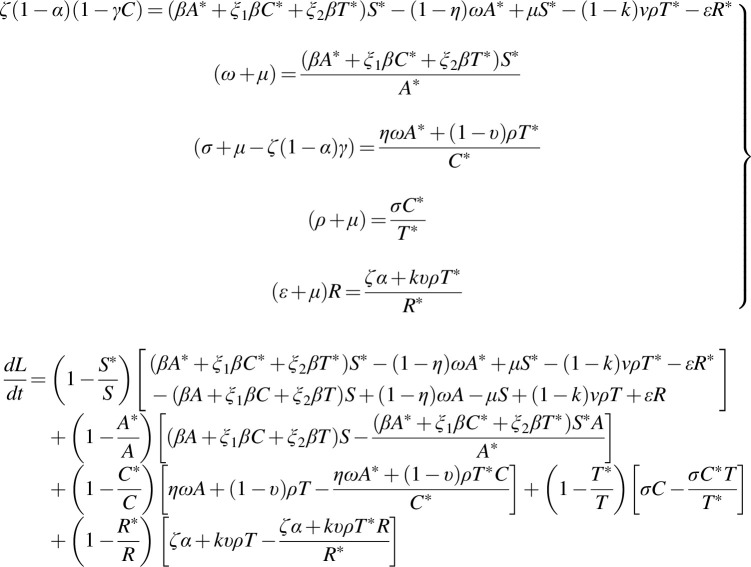

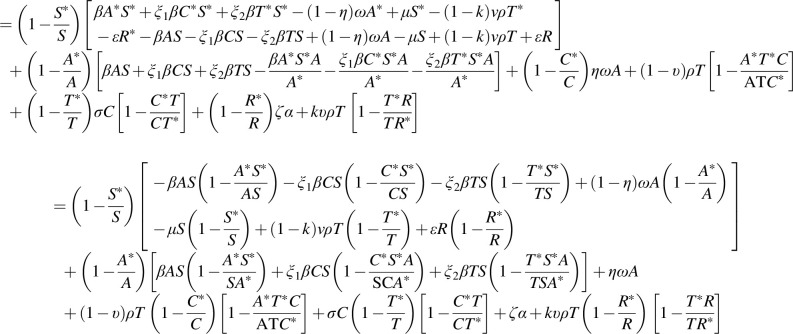

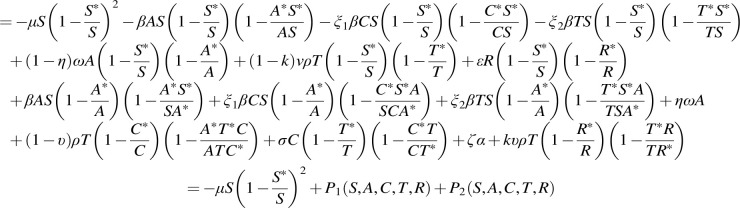


where,

**Figure:**
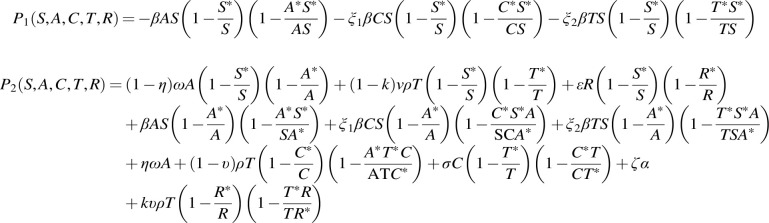


$P_{1}\leq 0$ whenever

(19) $$\mathrm{AS}\geq A^{*}S^{*},\mathrm{CS}\geq C^{*}S^{*},\mathrm{TS}\geq T^{*}S^{*}$$

and $P_{2}\leq 0$ whenever

**(20):**
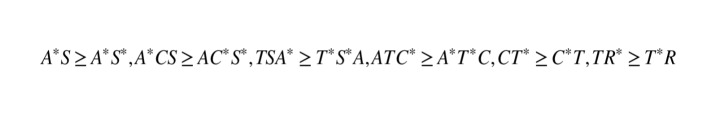


Thus,

$\frac{\mathrm{dL}}{\mathrm{dt}}\leq 0$ if the condition in (19) and (20) holds.

Therefore, by LaSalle asymptotic stability theorem ( LaSalle, 1976), and Adeniyi et al. (2020), the positive equilibrium state $\frac{\mathrm{dL}}{\mathrm{dt}}$ is globally asymptotically stable in the positive region $R_{+}^{5}$ .

### Sensitivity indices

To test the strength of the model and the parameter values, a sensitivity study was carried out. This is done in order know the parameters that have a huge influence on the basic reproduction number (R0) which is done using Maple 19 software. A variable k; a normalized forward sensitivity index which depends on a parameter: ℓ differentially, is defined as:

(21) $$\hbar_{\ell }^{k}=\frac{\partial k}{\partial \ell }\frac{\ell }{k}\;$$

The R0 sensitivity is therefore derived from each of the different parameters listed in Table 1. All expressions are complex for sensitivity indices, so sensitivity indices are evaluated in Table 2 at the baseline parameter values.

**Table 2.** Sensitivity indices on R0.

Parameter	Sensitivity index
ζ	1.0000041
β	0.9999999
ξ	0.0027767
ϵ	0.3456467
η	0.0027766
γ	0.0000004
σ	0.0026593
α	−0.054285
μ	−1.387192
ω	−0.955709

## Model validation

To validate our analytical results, we perform numerical simulations of the proposed model (2). These simulations are based on qualitative analysis. Some of the parameters were obtained from published research, while others were estimated by the authors as they were thought to be biologically feasible. We employ a strictly numerical RK (Runge-Kutta) technique of order four embedded in the Maple 19 software. Table 3 contains the parameter's comprehensive values.

**Table 3.** Parameter values used for the numerical simulation.

Parameter	Values	Source
ζ	0.012100	Khan et al (2019)
β	0.009500	Khan et al (2019)
ξ	0.160000	Khan et al (2019)
ϵ	0.050000	Estimated
η	0.067000	Estimated
γ	0.110000	Khan et al (2019)
σ	0.590000	Khan et al (2019)
α	0.320000	Khan et al (2019)
μ	0.006930	Khan et al (2019)
ω	0.160000	Estimated
k	0.300000	Estimated
ρ	0.005000	Estimated
υ	0.05000	Khan et al (2019)

**Figure 2.**
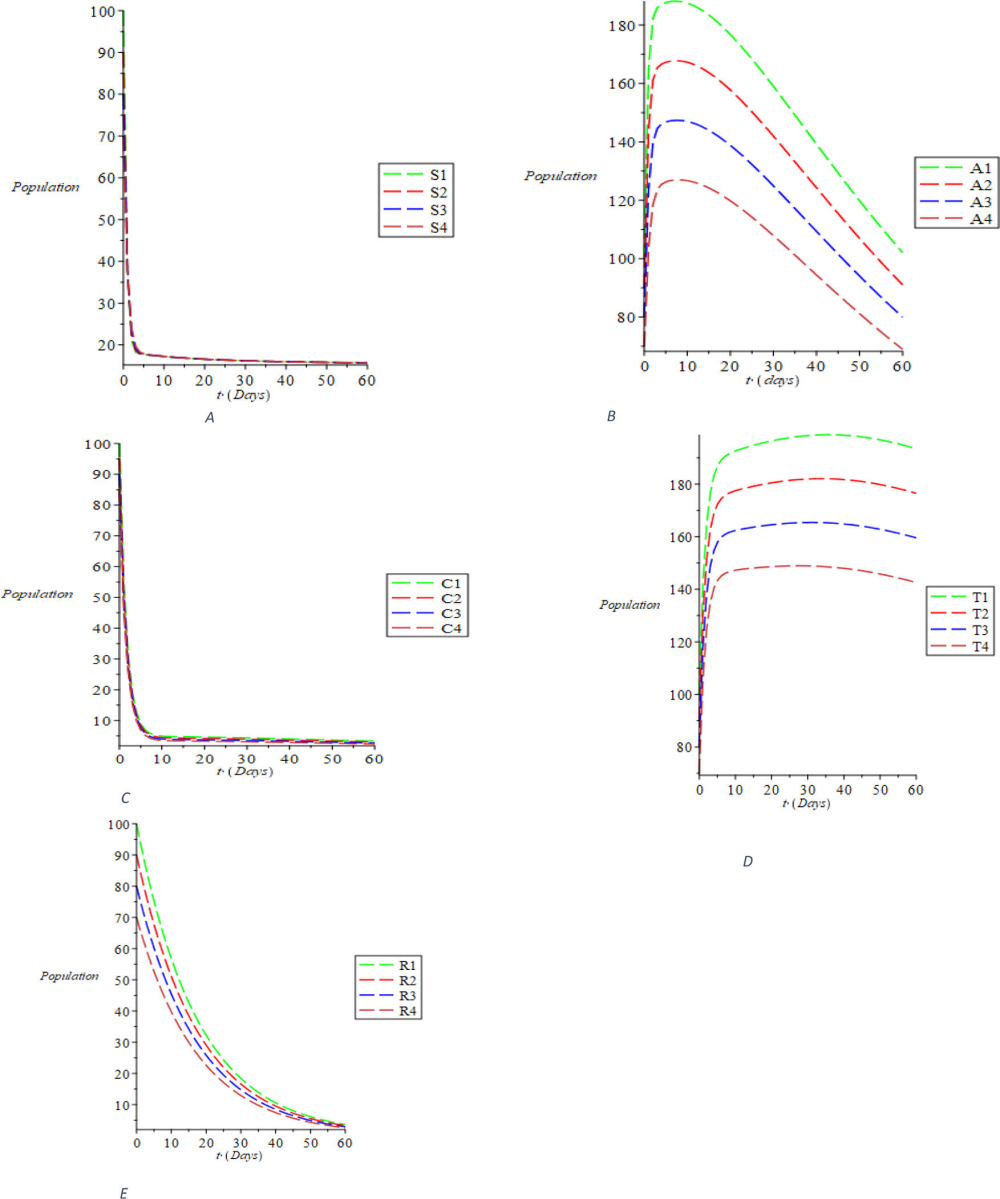
The dynamical behavior of the varying population of the classes: (A) susceptible (B) acute (C) chronic (D) treated (E) recovered using the Maple 19 software.

**Figure 3.**
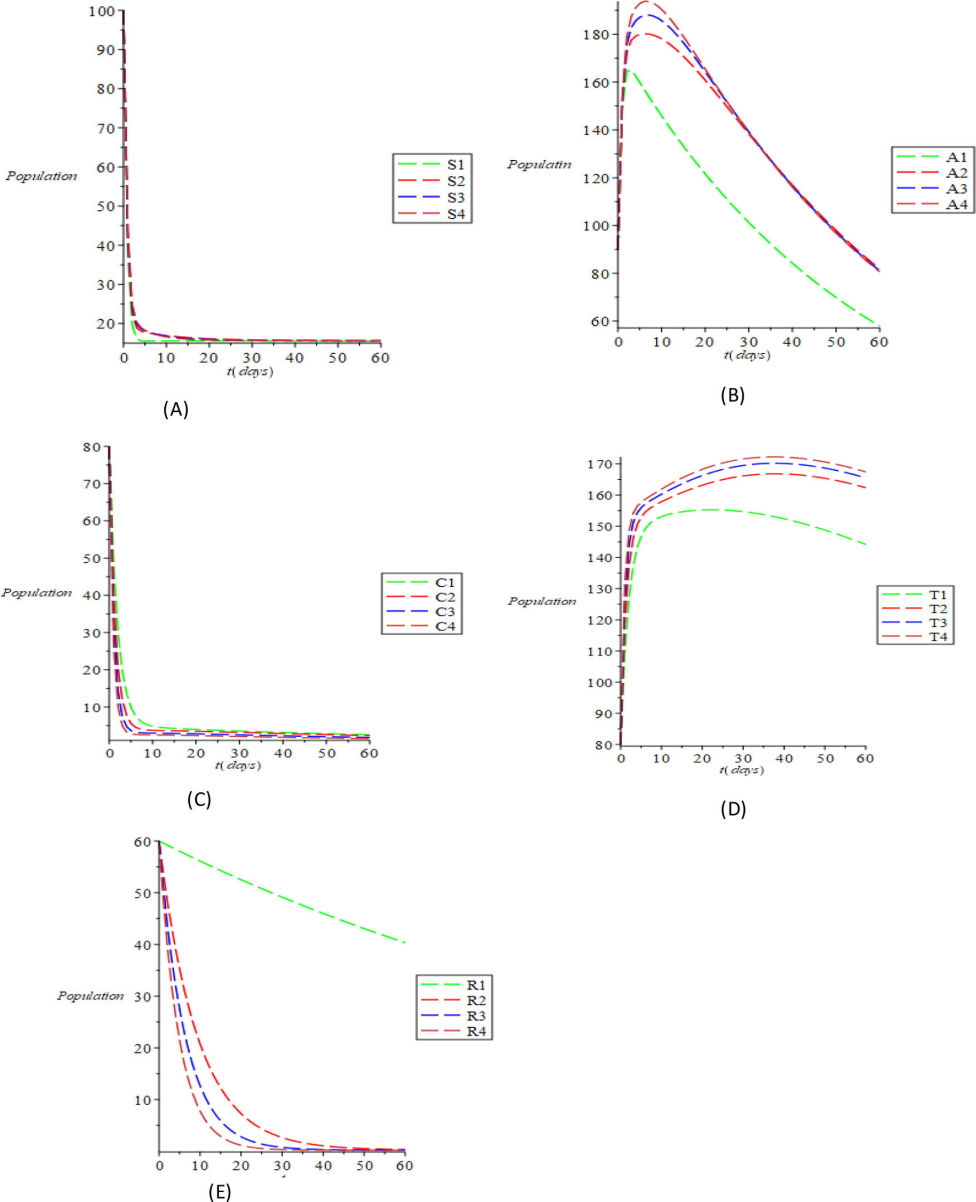
The dynamical behavior of the various classes varying various treatment parameter (A) susceptible (B) acute (C) chronic (D) treated (E) recovered using the Maple 19 software.

Considering the first sizes of compartmental population, taking the parameter values and the interval (0-60) using the linear stability analysis, we perform the simulations and obtain the outcomes shown in Figure 2. The dynamic behavior of susceptible individuals is represented in Figure 2A, showing the existence of the susceptible individuals. It was discovered from Figure 2B that the acute populace decreases with the passage of time. Ditto the behaviors of chronic, treated and recovered populace, respectively, are dynamically represented in Figure 2C, D and E. The trajectories S(t), A(t), C(t), T(t), and R(t) distinctly converge to the disease-free equilibrium of E0 = (S_0, 0, 0, 0, 0, R_0) = (1.678018396, 0, 0, 0, 0, 0, 0, 0.06801334973) as indicated in Eq. (10), when R0 = 0.07150316516< 1. The dynamics of the susceptible populace with respect to the treatment rate σ, recovering rate with full immunity k and the rate at which recovered individual fallout from risk reduction ϵ is shown in Figure 3A. It is evident from Figure 3A that with the increase in the parametric values, the susceptible population increases, even as increase is not evident due to those who recover with complete immunity. However, there is still an increase due to some people who fall out of risk reduction, checking the cumulative impact of the parameters causing the increment of the susceptible populations. The inverse relationship of the compartmental population (acute and chronic) with the variance of the above parameters is shown in Figure 3B and C. This means that it is possible to minimize acute and chronic individuals by increasing the parametric values. The variation of the treated and recovered populace is shown in Figure 3D and E. An increase in the treated and the recovered population is caused by increasing the values of the parameters. It can be clearly inferred from our computational simulations that treatment, spontaneous clearance and reduction of the risk factor are highly successful in transmitting and regulating HBV transmission. The effective measure of these parameters as substantiated by the simulations is an excellent control method of the transmissible infection of HBV.

## Conclusion

A deterministic model of hepatitis B that involves the spontaneous clearance of an acute individual and also recovery of chronic individual with full immunity and risk factor reduction was developed and investigated. Disease-free and endemic equilibria of the model exist. The basic reproduction number was constructed by the method of next generation matrix. The global stability of the disease-free and endemic equilibria was discussed and shown to be asymptotically stable. The effects of the treatment rate, the recovery rate with complete immunity, and the risk mitigation factor were thoroughly discussed. Future work may include using the optimum control theory to mitigate hepatitis B infection.

## Data availability

All data underlying the results are available as part of the article and no additional source data are required.
