# Supplementary material for: Global stability analysis of hepatitis B virus dynamics
Source: F1000Res. 2022 Jan 21;10:429. Originally published 2021 May 28. [Version 2] doi: 10.12688/f1000research.52785.2 (PMC9043681; doi:10.12688/f1000research.52785.2)
Supplement: Peer review history

# Peer review: Reviewer response for version 2

**DOI:** 10.5256/f1000research.78507.r135850

**Published:** 2022-05-06

**Authors:** Syafruddin Side

**Affiliations:** 1 Department of Statistics, Faculty of Mathematics and Natural Science, Universitas Negeri Makassar, Makassar, Indonesia

Comment result: 

1. Each equation in the system should be assigned an equation number
2. In proving each lemma and theorem, it is expected to provide more information or references that strengthen the proof steps
3. The equilibrium point cannot be negative, so it is necessary to improve the way of writing the endemic equilibrium point so that it does not appear negative
4. Matrices F and V are obtained using which equation? Please clarify
5. Each image from the simulation should be explained and interpreted clearly so that it can be more easily understood by the reader
6. References used should be published in a maximum of 5 years
7. Add references so that it strengthens the research results
8. The research method used is not clear so it needs to be explained briefly and concisely

Is the work clearly and accurately presented and does it cite the current literature?

Yes

If applicable, is the statistical analysis and its interpretation appropriate?

Yes

Are all the source data underlying the results available to ensure full reproducibility?

Yes

Is the study design appropriate and is the work technically sound?

Yes

Are the conclusions drawn adequately supported by the results?

Yes

Are sufficient details of methods and analysis provided to allow replication by others?

Partly

Reviewer Expertise:

Modeling Mathematics

I confirm that I have read this submission and believe that I have an appropriate level of expertise to confirm that it is of an acceptable scientific standard, however I have significant reservations, as outlined above.

# Peer review: Reviewer response for version 2

**DOI:** 10.5256/f1000research.78507.r135851

**Published:** 2022-04-26

**Authors:** Ran Zhang

**Affiliations:** 1 Department of Mathematics, Nanjing University of Aeronautics and Astronautics, Nanjing, China

This study focus on a hepatitis B virus model. The existence and the positivity solution of the model are established. The stability analysis based on the basic reproduction number are obtained. Finally, computational simulations are presented.

 Overall, the mathematical analysis in the current paper is standard, but there are many gaps in the proof. I suggest that the authors should modify the paper according to the following suggestions and comments:

1. The expression of the article needs to be improved, there are lots of mistakes in writing and grammar. For example, “classes” should be “class” in Line 8 of Page 4, “R(0)\geq ,0” should be “R(0)\geq0”. There are so many errors and the author should check the whole paper carefully.
2. Page 4, Line 10-15, the authors give the biological significance of parameters for the model., the authors should delete these to avoid duplication because the biological significance of parameters are also given in Table 1.
3. Page 5, Line 9, please delete the definition of $\lambda_S$ since it has already defined in Equation (2).
4. What is the meaning of $\Phi$ in Lemma 1, please give the definition. This question was also proposed by the previous reviewer, but the author did not answer it.
5. The expressions of proving for each theorem need to be improved, for example, Line 5-6 of Page 6, there is no conjunction between the two equations. This problem also happened in the rest of this paper.
6. Brackets should not appear in Equation (5)-(9). For example, “R^* = (H/L)” should be “R^* = H/L”.
7. The authors said they have used next generation matrix theorem, but there is no literature for this theory. I think the authors should add some references.
8. Line 14 of Page 7, “reproduction number” should be “basic reproduction number”.
9. Page 7, Equation (10), “R0” should be $R_0$ and this problem happens in the rest of this paper.
10. Page 8, Equation (11), the eigenvalues are represented by $\lambda$, but this letter is already represented the birth rate in the model, please use another letter.
11. I don't understand the logic of the last line of Page 8. Does this means that $R_0<1$ is a conclusion? But as I can see, $R_0<1$ is the condition of Theorem 1.
12. Page 9, Theorem 2, there is no proof for the positive equilibrium.
13. At the last part of the proof for Theorem 2, there are some additional conditions (19) and (20). But I can not see conditions (19) and (20) in Theorem 2, and these conditions are very strong mathematically.

Although there are some gaps in the mathematical analysis of this article, I think the conclusion should be correct. The author only needs to improve the process of mathematical analysis.

Is the work clearly and accurately presented and does it cite the current literature?

Yes

If applicable, is the statistical analysis and its interpretation appropriate?

Not applicable

Are all the source data underlying the results available to ensure full reproducibility?

Yes

Is the study design appropriate and is the work technically sound?

Yes

Are the conclusions drawn adequately supported by the results?

Yes

Are sufficient details of methods and analysis provided to allow replication by others?

Partly

Reviewer Expertise:

Mathematical Biology

I confirm that I have read this submission and believe that I have an appropriate level of expertise to confirm that it is of an acceptable scientific standard.

# Peer review: Reviewer response for version 1

**DOI:** 10.5256/f1000research.56106.r90565

**Published:** 2021-08-16

**Authors:** Abimbola Abolarinwa

**Affiliations:** 1 Department of Mathematics, University of Lagos, Lagos, Nigeria

The authors develop and investigate the dynamic of HBV-model incorporating spontaneous clearance of acute individuals and recovery pf chronic individuals with full immunity. The model is subjected to sensitivity and stability analysis.

 The results are well presented and discussed. I can recommend this paper for indexing if the authors can consider these suggestions:

1. Under Abstract - change positivity solution to positivity of solutions
2. Under Introduction - line 3 - change reporting to reported. Paragraph 4 - should be put in proper perspective to show the gap which this paper is trying to bridge. In my understanding, testing and treatment have always been part of epidemic (including HBV) control. You can advertise the novelty introduced in the paper. The statement "The aim of this study is to contribute ....." can be recast to make the aim measurable and specific to indicate what and what is being contributed and the its importance.
3. Under model implementation.

Lemma 1 -

- Start the statement with "Suppose the initial values are ..." No need for the word parameters
- No need for $\in \Phi$ or $\Phi$ should be defined
- The solution of the model (3) {...} is posively invariant for all t>0. (not greater than or equal to).

Under the proof

 The last three line on page 4(so also the first 4 lines on page 5) should be corrected

 These two lines can help in each case

 "we have

 $$\frac{dS}{dt} \ge -(\lamda_s+\mu)S$$

 and

 $$S(t)\ge s_0e^{-\lambda+\mu)t.$$ (t should be included in power of e)

 where $s_0=S(0)\ge 0$.

 Hence S(t)> 0 for all t>0.

Is the work clearly and accurately presented and does it cite the current literature?

Yes

If applicable, is the statistical analysis and its interpretation appropriate?

Not applicable

Are all the source data underlying the results available to ensure full reproducibility?

Partly

Is the study design appropriate and is the work technically sound?

Yes

Are the conclusions drawn adequately supported by the results?

Yes

Are sufficient details of methods and analysis provided to allow replication by others?

Yes

Reviewer Expertise:

Differential equations, Differential Geometry

I confirm that I have read this submission and believe that I have an appropriate level of expertise to confirm that it is of an acceptable scientific standard.

# Response

**Published:** 2021-10-15

**Authors:** Olajumoke Oludoun

**Affiliations:** LANDMARK UNIVERSITY, Nigeria

All the comments have been attended to in the new version submitted
